# Supplementary material for: Generating property-matched decoy molecules using deep learning
Source: Bioinformatics. 2021 Feb 3;37(15):2134–41. doi: 10.1093/bioinformatics/btab080 (PMC8352508; doi:10.1093/bioinformatics/btab080)
Supplement: btab080_Supplementary_Data [file btab080_supplementary_data.zip › SI_Generating_Property-Matched_Decoy_Molecules_Using_Deep_Learning.pdf]

Supplementary Information
Generating Property-Matched Decoy Molecules Using Deep Learning

Fergus Imrie,† Anthony R. Bradley,‡ and Charlotte M. Deane*,†

†*Oxford Protein Informatics Group, Department of Statistics, University of Oxford, Oxford OX1 3LB, UK*

‡*Exscientia Ltd, 36 St. Giles', Oxford, OX1 3LD, UK*

E-mail: deane@stats.ox.ac.uk

Additional DeepCoy model details

Atom types.

In line with both Liu et al.[1] and Imrie et al.[2], 14 atom types are permitted in the standard version of our model: carbon, nitrogen (N^-, N, N^+), oxygen (O^-, O, O^+), fluorine, chlorine, bromine, iodine, and sulphur (maximum valence 2, 4, or 6).

All active molecules for DUD-E target FPPS contained phosphorus, and thus for this target we included phosphorus as an additional atom type (with maximum valence 5).

Phosphorus training set.

The 250 000 molecule subset of ZINC[3] selected at random by Gómez-Bombarelli et al.[4] used to train the standard versions of DeepCoy contains only 123 compounds with phosphorus. In order to construct a more suitable training set, we extracted all phosphorus-containing compounds from ZINC[3] and followed the same procedure and thresholds described in Section 2.2 to construct a training set. This process resulted in 109,061 pairs that were used to train our model. We used identical hyperparameters as the other versions of DeepCoy.

Network architecture.

Following Liu et al.[1], both the encoder and decoder utilise standard gated graph neural networks (GGNN),[5] which propagate messages for 7 steps and have residual connections between odd numbered time steps.

The neural network mapping the hidden state of a node to its atom type is implemented as a linear classifier, following Liu et al.[1].

Hyperparameters.

We trained the model with a learning rate of 0.001 for 10 epochs using the Adam optimiser and a batch size of 8, selecting the model from the epoch with the lowest validation loss. The dimension of the latent space was 100 and the dimension of the vector used to encode the target molecule during training and sampled from a $\mathcal{N}(\mathbf{0}, \boldsymbol{I})$ distribution when generating new molecules was 8. λ_{KL} was set at 0.3.

We performed limited hyperparameter optimisation. However, we found that the model required a larger latent space dimension than in Imrie et al.[2], with the dimension of the latent space matching Liu et al.[1]. We believe this is due to the additional number of steps required to construct a full molecule compared to a partial structure as well as the importance of the input molecule for the generation task.

Physicochemical properties to unbias

This section contains a list of properties that were unbiased. We calculated the values for all properties using RDKit.[6] This will likely cause minor discrepancies with the property values calculated in the construction of both DUD-E[7] and DEKOIS 2.0.[8] Indeed, values of the metrics used to assess property-matching and structural similarity were similar but not identical to the values reported by Bauer et al.[8]. All values reported here are calculated in the same way and thus directly comparable.

DUD-E.

DUD-E[7] selected the following six properties to match: molecular weight, log P, number of hydrogen bond acceptors, number of hydrogen bond donors, number of rotatable bonds, and net charge.

DEKOIS 2.0.

DEKOIS 2.0[8] was constructed by matching the following eight properties: molecular weight, log P, number of hydrogen bond acceptors, number of hydrogen bond donors, number of rotatable bonds, number of aromatic rings, positive charge, and negative charge.

All properties.

In our experiments using the DUD-E data set, we trained our model to match a substantially larger number of properties to assess whether DeepCoy could handle higher-dimensional restrictions.

Chaput et al.[9] focused their analysis of the DUD-E data set on nine properties, largely overlapping with the original DUD-E properties. In particular, they noted deviation between the DUD-E actives and decoys for the embranchment count and polar surface area. We included these properties as the number of chiral centers (Chaput et al.[9] reported a correlation of 0.97 with embranchment count) and topological polar surface area (TPSA).

MUV[10] was constructed to unbias the following 17 properties: simple counts of all atoms, heavy atoms, boron, bromine, carbon, chlorine, fluorine, iodine, nitrogen, oxygen, phosphorus, and sulfur atoms, number of hydrogen bond acceptors, number of hybond donors, logP, number of chiral centers, and number of rings.

Combining the properties used to construct DUD-E, DEKOIS 2.0, and MUV, together with the two additional properties from Chaput et al.[9], synthetic accessibility,[11] and quantitive estimation of druglikeness (QED)[12] yields 27 unique physicochemical properties.

Deep learning-based SBVS

As further validation, we used the DeepCoy decoys to both train and test deep learning-based SBVS methods. Specifically, we used the DUD-E set with both the original decoys and the DeepCoy decoys and trained the convolutional neural networks proposed by Ragoza et al.[13]

("gnina") and Imrie et al.[14] ("DenseU" and "DenseFS"). For an external set, we used the subset of the datasets curated from ChEMBL ([15]) by Riniker and Landrum[16] adopted by Ragoza et al.[13]. This subset was selected by Ragoza et al.[13] to ensure that models were evaluated on targets dissimilar to those in the training set. This was achieved by ensuring a maximum global sequence similarity of 80% with any training target and also removing any target which had a significant structural alignment of its binding site with any training target according to ProBiS[17] (using the default ProBiS parameters).

As discussed in Section 3.3, the ChEMBL targets share similar biases to the original DUD-E, but shared limited bias with the DeepCoy decoys. As such, it is not appropriate to compare the performance of models trained or evaluated on the DUD-E and DeepCoy decoys. In addition, limited conclusions about the relative quality of the decoy molecules can be made based on these experiments as it is challenging to conclude if models trained on DUD-E have learnt bias or meaningful features.

Implementation details. Following Ragoza et al.[13] and Imrie et al.[14], we trained the CNN models only on the top-ranked AutoDock Vina pose for each complex. Models were trained using stochastic gradient descent with a learning rate of 0.01, momentum of 0.9, and weight decay of 0.001. Models were trained for the same number of iterations with the same batch sizes described in Ragoza et al.[13] and Imrie et al.[14]. We employed the same data augmentation scheme as Ragoza et al.[13]. We did not perform any hyperparameter tuning. We trained three replicas of the models using different random seeds. The performance of the random seeds was averaged in the case of gnina and DenseU, while the predictions were combined in an ensemble for DenseFS. Models were implemented using PyTorch[18] and libmolgrid[19] for molecular gridding and code can be found at https://github.com/oxpig/DenseFS.

Train DUD-E, Test ChEMBL. Table S1 shows the performance of AutoDock Vina, gnina[13] and DenseU[14] on the ChEMBL test sets trained on the original version of DUD-E and the version of DUD-E employing DeepCoy decoys.

While there is a significant reduction in the predictive power of the CNN-based methods

when trained on DeepCoy decoys, a random forest model trained on the unbiased features experiences a larger decrease in performance (average AUC ROC all features: DUD-E 0.84, DeepCoy 0.57).

As discussed in Section 3.3, due to the shared bias in the original DUD-E dataset and ChEMBL, it is challenging to conclude whether models have learnt bias or meaningful features. However, since the version of DUD-E employing DeepCoy decoys does not contain these biases, we can be more confident that any predictive power on the ChEMBL test set arises from learning meaningful features. When trained on the version of DUD-E employing DeepCoy decoys, the CNN-based models outperform both AutoDock Vina and the random forest model trained on the unbiased features.

Train ChEMBL, Test DUD-E. We trained the same CNN models using the ChEMBL targets and rescored the docked poses of the DUD-E set. In line with AutoDock Vina, gnina had lower virtual screening performance when assessed on the DeepCoy decoys compared to the original decoy molecules. However, while DenseU experienced a marginal drop in performance as measured by average AUC ROC, early enrichment and AUC PRC saw modest increases (Table S2).

Notably, AutoDock Vina experienced the largest fall in predictive performance, despite being a linear combination of five energy terms representing protein-ligand interactions and the number of rotatable bonds in the ligand. The virtual screening performance as measured by AUC ROC fell by 0.07 from 0.70 to 0.63 compared to smaller drops for gnina (reduction of 0.05) and DenseU (reduction of 0.02). This was also true for the other performance metrics.

In addition, we generated DeepCoy decoys for the ChEMBL test sets. The average DOE score for the ChEMBL test targets was 0.173 and 0.170, calculated using the original DUD-E properties or all properties, respectively. Using DeepCoy decoys the average DOE scores fell to 0.033 and 0.026, respectively, indicating a substantial reduction in bias.

We repeated the above experiment, replacing the original decoys in the ChEMBL test sets with DeepCoy decoys and trained CNN-based methods on these sets. As expected,

the performance of both CNN-based methods when trained on DeepCoy decoys fell for the original DUD-E set, since the shared bias between the training and test set has been removed (Table S3). The performance on the DeepCoy version of DUD-E improved, likely due to the mismatch of unbiased properties being removed and the lack of independence between decoys in the training and test set. This suggests that even when decoys exhibit minimal bias on a per-target basis, independent test sets should be employed for validation, in line with accepted practice for model evaluation (e.g.[20–22])

Due to the limited training set size (only 14 protein targets with 100 actives per target) and the bias shared between the ChEMBL targets and the original DUD-E set, we believe that limited conclusions can be drawn from these experiments.

Additional results

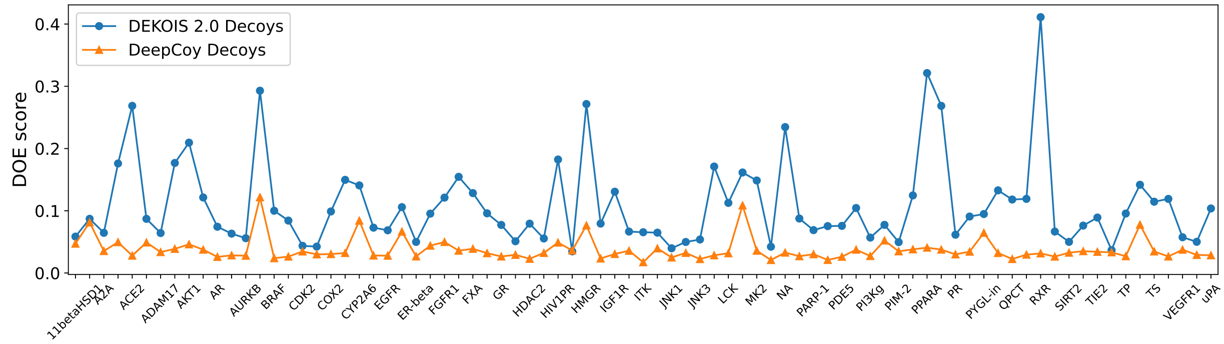

Figure S1: DOE scores of the original DEKOIS 2,0 set (blue) compared to the DeepCoy generated decoys (orange). The x-axis displays each DEKOIS 2.0 target in the same order as they appear in the DEKOIS 2.0 database (http://www.dekois.com/). The targets with even indices are not labeled on the x-axis due to space limitations.

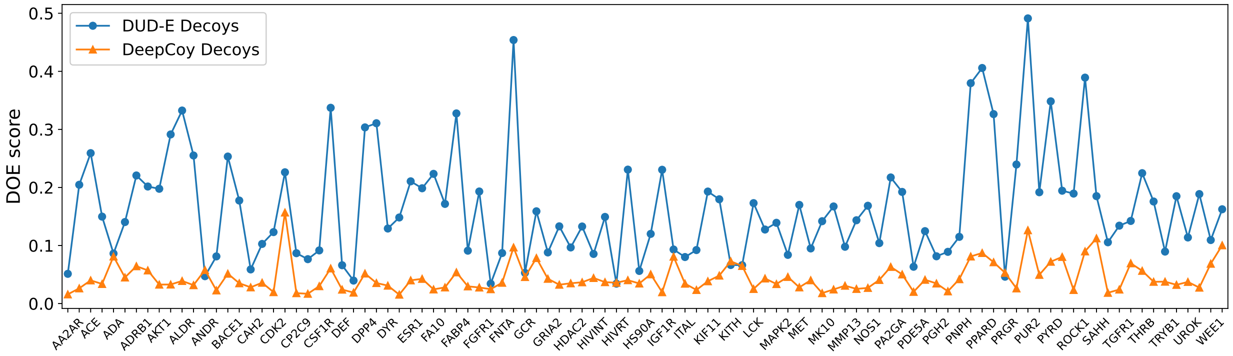

Figure S2: DOE scores of the original DUD-E set (blue) compared to the final DeepCoy generated decoys (orange) that were selected based on a larger number of properties to unbias, calculating DOE score using only the original DUD-E properties. The x-axis displays each DUD-E target in the same order as they appear in the DUD-E database (http://dude.docking.org/targets). The targets with even indices are not labeled on the x-axis due to space limitations.

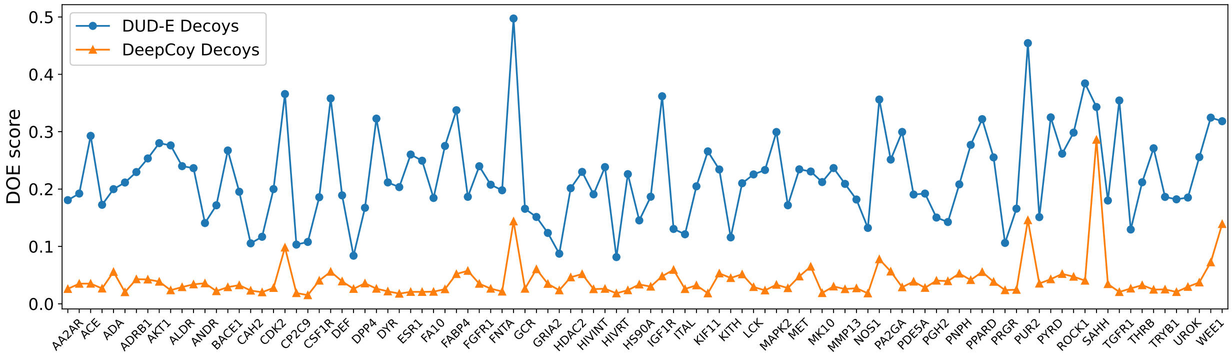

Figure S3: DOE scores of the original DUD-E set (blue) compared to the final DeepCoy generated decoys (orange) that were selected based on a larger number of properties to unbias, calculating DOE score using all 27 properties to unbias. The x-axis displays each DUD-E target in the same order as they appear in the DUD-E database (http://dude.docking.org/targets). The targets with even indices are not labeled on the x-axis due to space limitations.

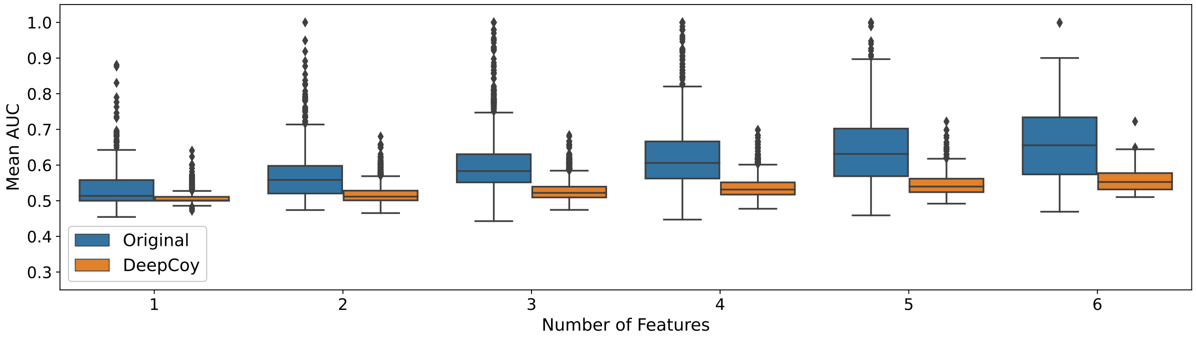

Figure S4: Results of the machine learning-based assessment of physicochemical property matching on DUD-E. A 1-nearest neighbour model was trained to predict whether a compound was an active or a decoy based on the unbiased features. Virtual screening performance was assessed by AUC ROC for the original DUD-E decoys and DeepCoy generated decoys. The DeepCoy generated decoys resulted in a reduction in the median per-target AUC ROC using all 6 features from 0.66 to 0.55, indicating a substantial reduction in bias.

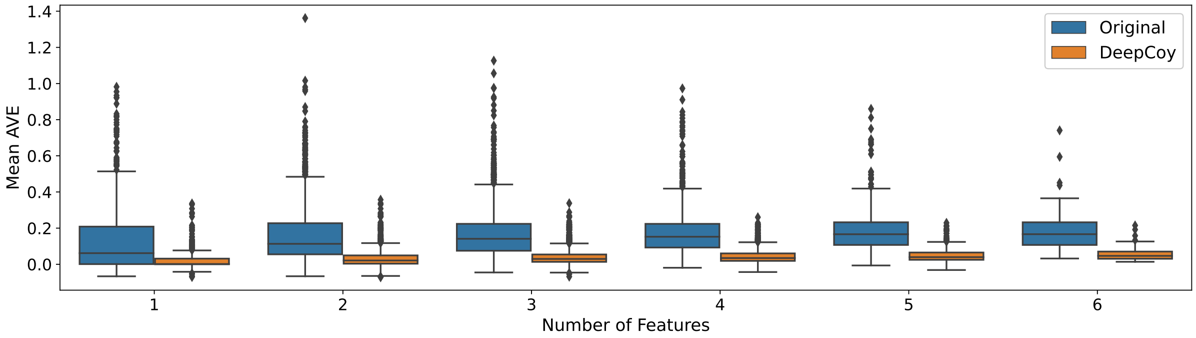

Figure S5: Results of the AVE assessment of physicochemical property matching on DUD-E. The DeepCoy generated decoys resulted in a 72% reduction in the median AVE using all 6 features decreasing from 0.17 to 0.05, indicating a substantial reduction in bias.

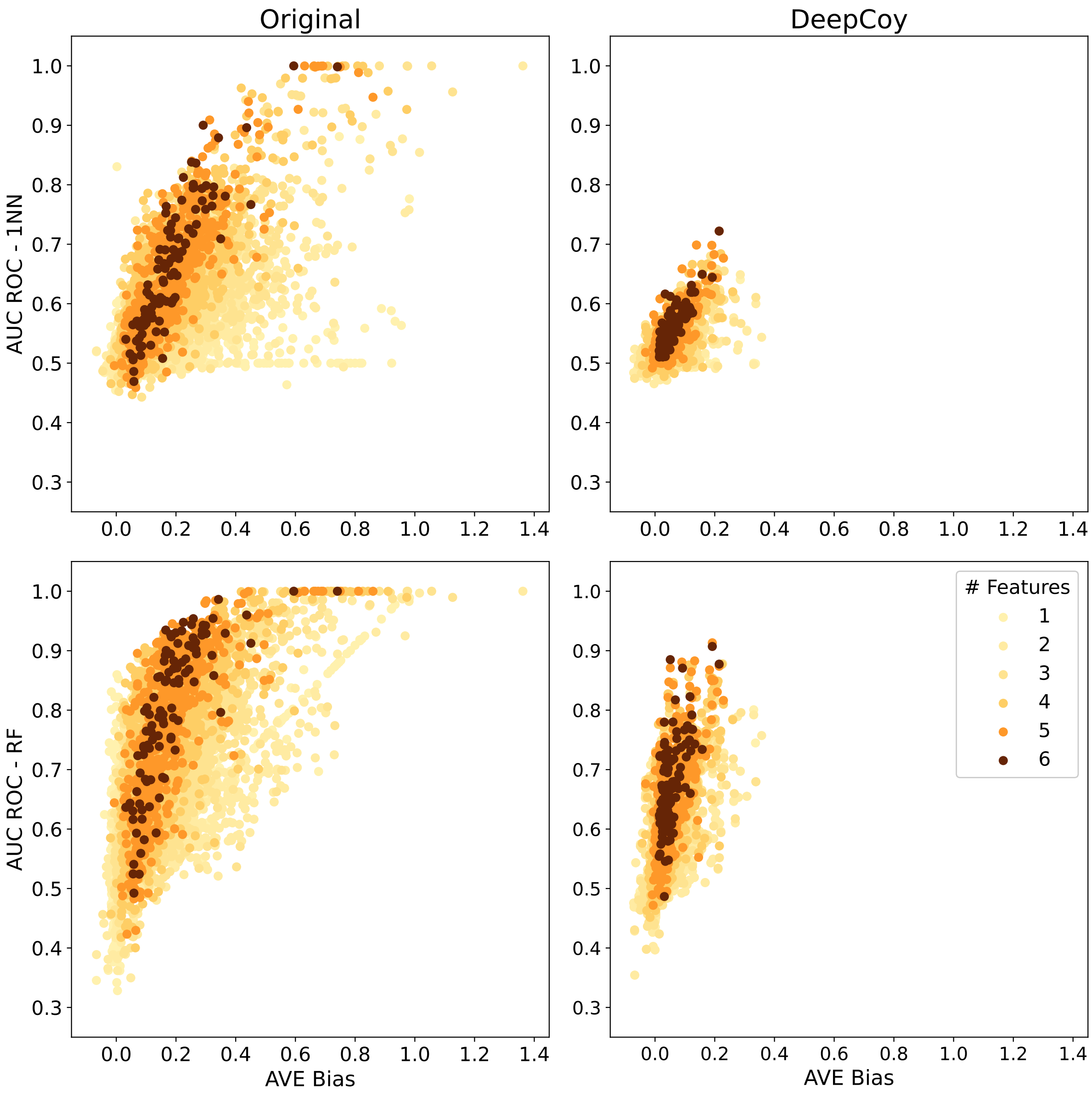

Figure S6: Comparison of machine learning-based assessment and AVE assessment of physicochemical property matching on DUD-E.

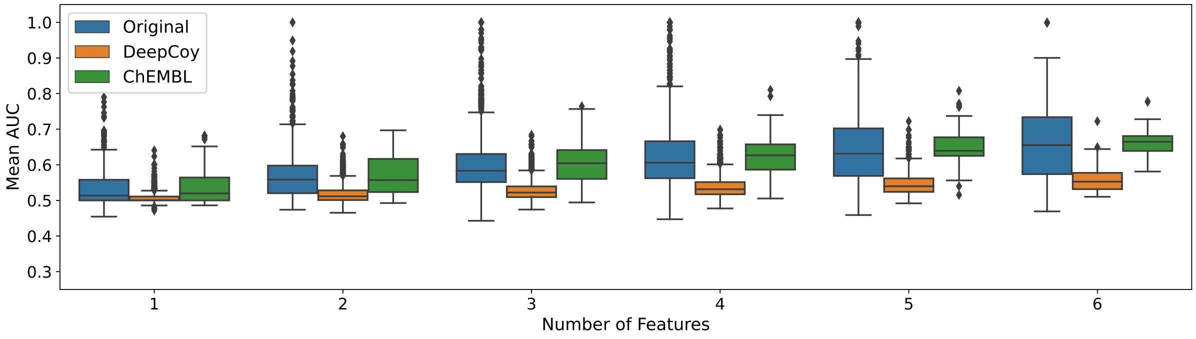

Figure S7: Results of the machine learning-based assessment of physicochemical property matching on DUD-E and the ChEMBL test sets. A 1-nearest neighbour model was trained to predict whether a compound was an active or a decoy based on the unbiased features. Virtual screening performance was assessed by AUC ROC.

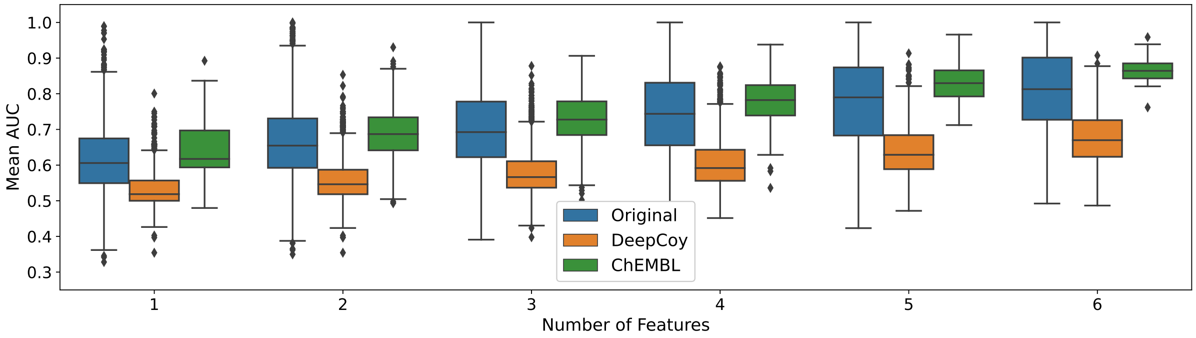

Figure S8: Results of the machine learning-based assessment of physicochemical property matching on DUD-E and the ChEMBL test sets. A 1-nearest neighbour model was trained to predict whether a compound was an active or a decoy based on the unbiased features. Virtual screening performance was assessed by AUC ROC.

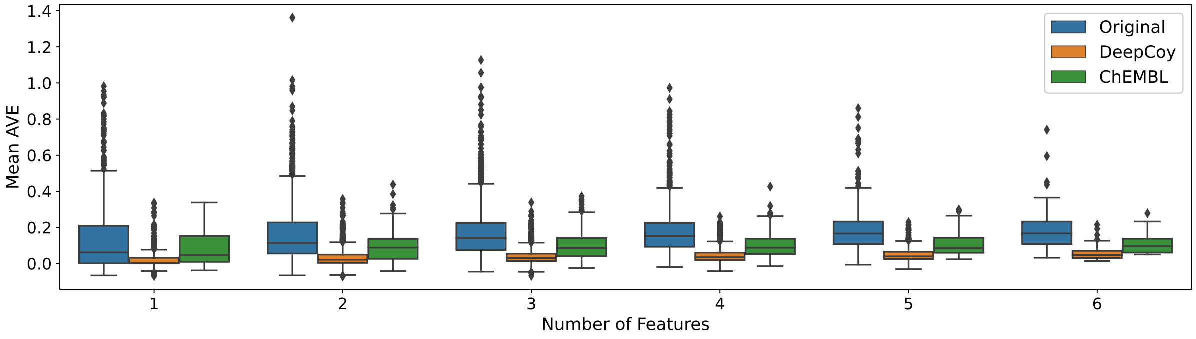

Figure S9: Results of the AVE assessment of physicochemical property matching on DUD-E and the ChEMBL test sets.

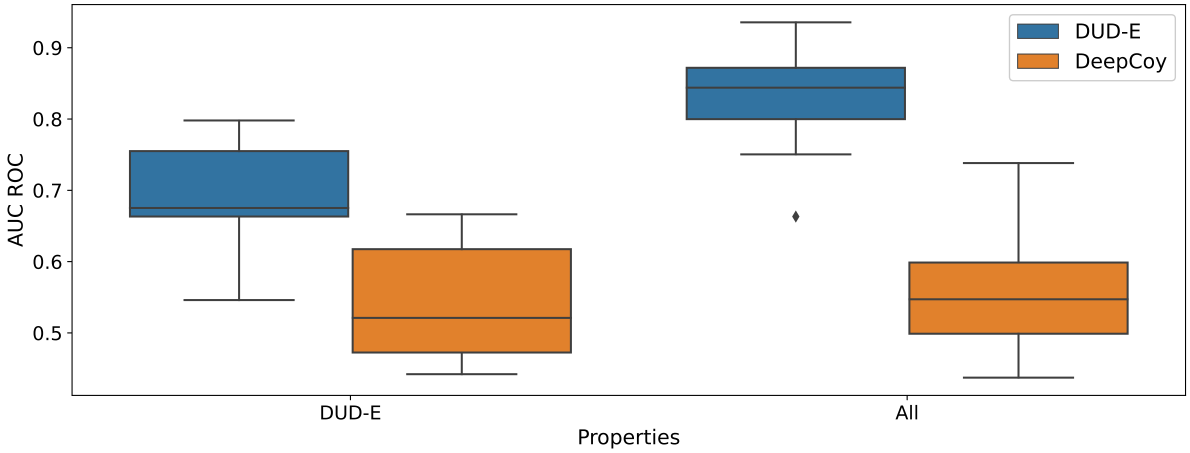

Figure S10: Results of the machine learning-based assessment of physicochemical property matching on the ChEMBL test sets. A random forest model was trained on the DUD-E targets to predict whether a compound was an active or a decoy based on the unbiased features. Virtual screening performance was assessed by AUC ROC.

Table S1: SBVS performance on ChEMBL test sets when trained on DUD-E with either the original or DeepCoy decoys. We used the smina[23] implementation of AutoDock Vina.[24] "gnina" refers to the CNN model of Ragoza et al.[13] and "DenseU", "DenseFS" to the universal and protein family-specific CNN models of Imrie et al.[14], respectively.

	AutoDock	gnina		DenseU		DenseFS	
Metric	Vina	Original	DeepCoy	Original	DeepCoy	Original	DeepCoy
AUC ROC	0.66	0.79	0.74	0.80	0.76	0.82	0.77
AUC PRC	0.04	0.14	0.07	0.17	0.07	0.21	0.11
0.5% ROC EF	9.29	30.29	14.77	36.97	14.01	44.02	23.58
1.0% ROC EF	7.51	21.34	11.01	23.56	11.20	27.37	16.01
2.0% ROC EF	5.32	14.47	7.92	15.33	8.71	17.69	10.26
5.0% ROC EF	4.29	8.42	5.36	8.23	5.99	9.35	6.35

Table S2: SBVS performance on DUD-E with either the original or DeepCoy decoys when trained on the ChEMBL test sets. We used the smina[23] implementation of AutoDock Vina.[24] "gnina" refers to the CNN model of Ragoza et al.[13] and "DenseU" to the universal CNN model of Imrie et al.[14].

	AutoDock Vina		gnina		DenseU	
Metric	Original	DeepCoy	Original	DeepCoy	Original	DeepCoy
AUC ROC	0.70	0.63	0.77	0.72	0.75	0.73
AUC PRC	0.09	0.06	0.08	0.07	0.07	0.09
0.5% ROC EF	15.02	8.54	7.57	6.88	8.37	12.60
1.0% ROC EF	10.39	6.36	6.92	6.11	7.01	9.90
2.0% ROC EF	7.14	4.72	6.26	5.42	5.72	7.52
5.0% ROC EF	4.73	3.21	5.14	4.39	4.32	5.32

Table S3: SBVS performance on DUD-E with either the original or DeepCoy decoys when trained on the ChEMBL test sets with the original decoys replaced with DeepCoy decoys. We used the smina[23] implementation of AutoDock Vina.[24] "gnina" refers to the CNN model of Ragoza et al.[13] and "DenseU" to the universal CNN model of Imrie et al.[14].

	AutoDock Vina		gnina		DenseU	
Metric	Original	DeepCoy	Original	DeepCoy	Original	DeepCoy
AUC ROC	0.70	0.63	0.70	0.82	0.70	0.84
AUC PRC	0.09	0.06	0.04	0.14	0.05	0.21
0.5% ROC EF	15.02	8.54	3.49	19.56	6.46	35.91
1.0% ROC EF	10.39	6.36	3.32	14.50	5.26	24.61
2.0% ROC EF	7.14	4.72	3.23	10.99	4.45	16.39
5.0% ROC EF	4.73	3.21	2.83	7.28	3.56	9.14

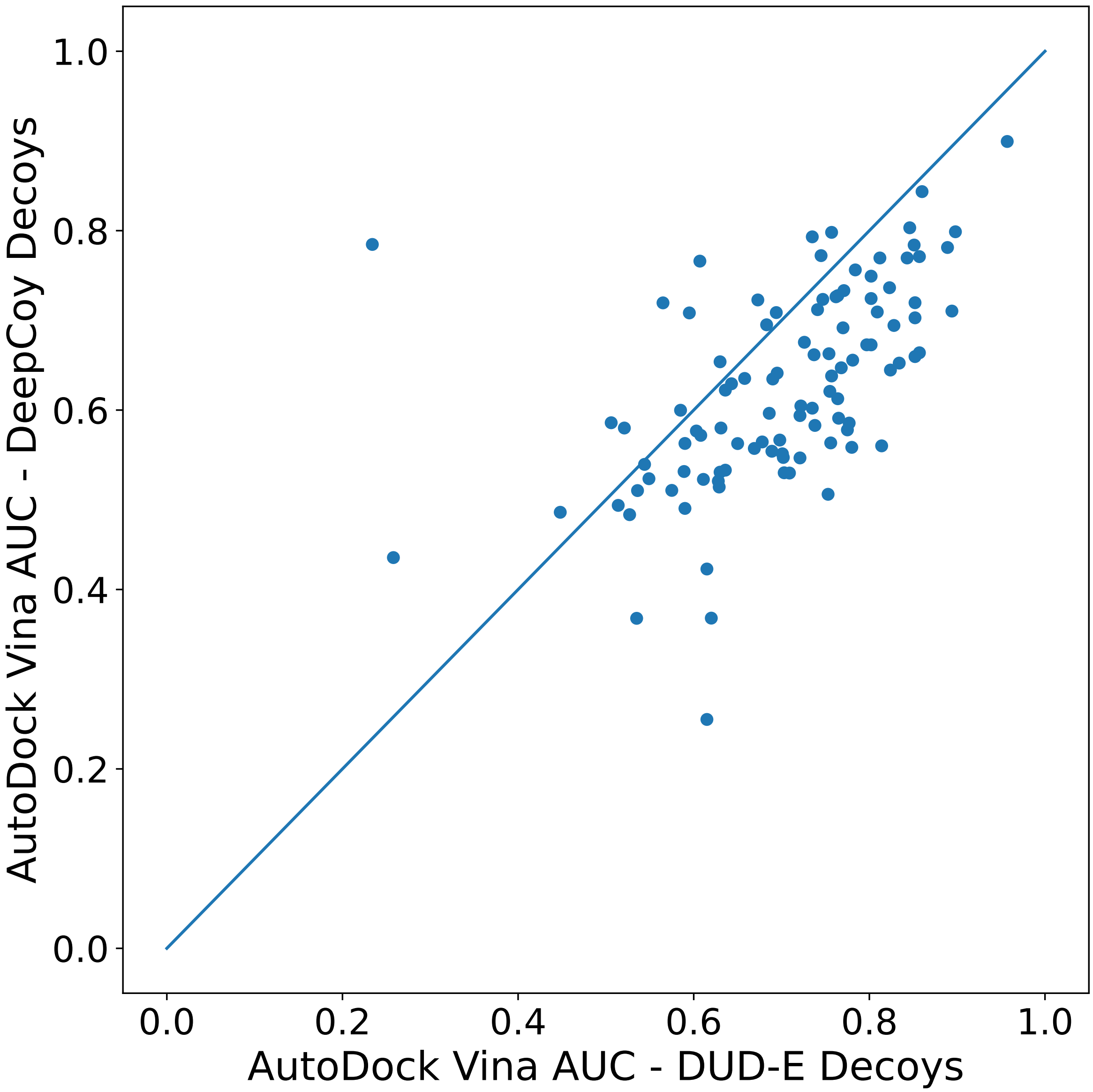

Figure S11: Virtual screening performance of docking using AutoDock Vina as measured by AUC ROC. The average AUC ROC decreased from 0.70 for the original DUD-E set to 0.63 for the DeepCoy generated decoys.

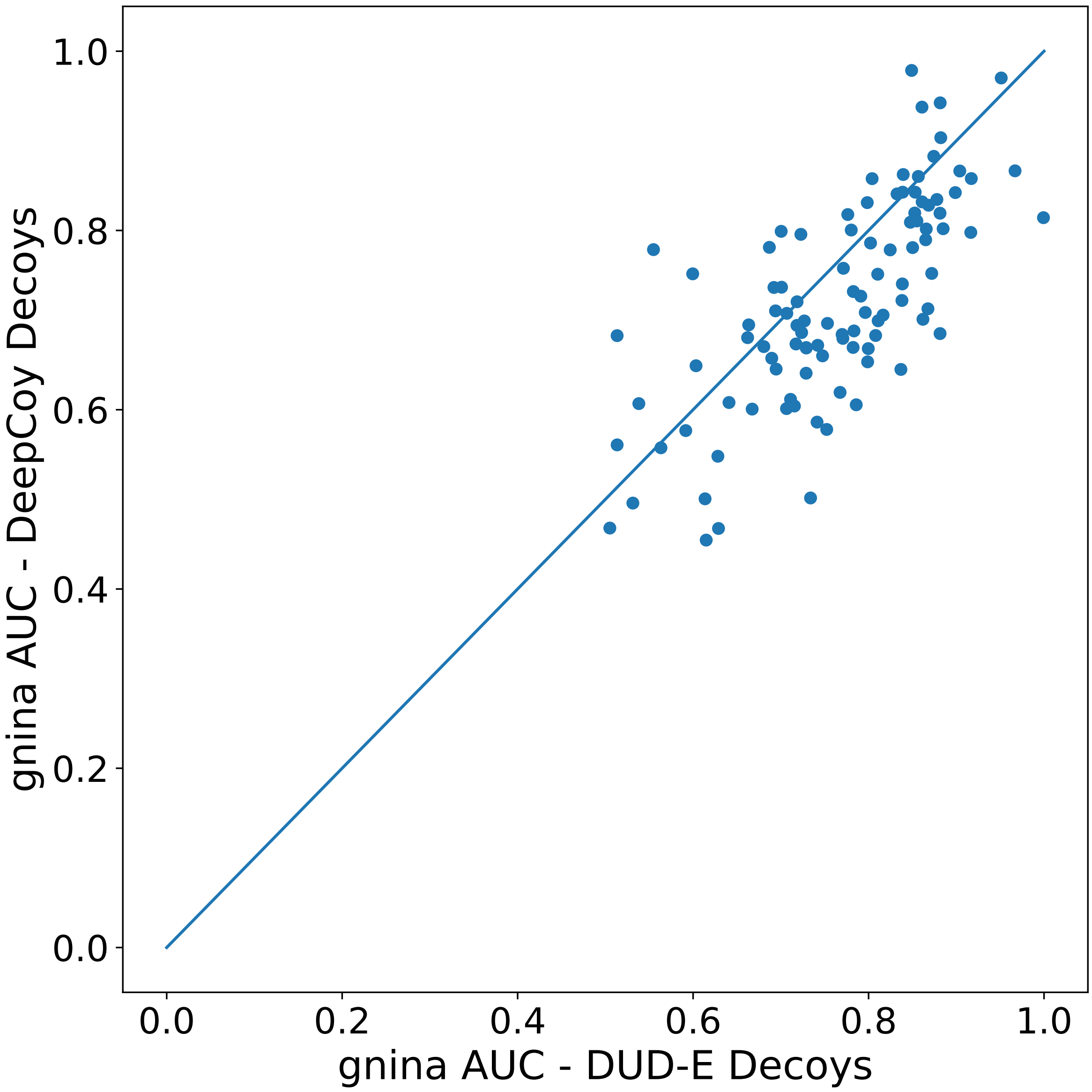

Figure S12: Virtual screening performance of gnina[13] as measured by AUC ROC. The average AUC ROC decreased from 0.77 for the original DUD-E set to 0.72 for the DeepCoy generated decoys.

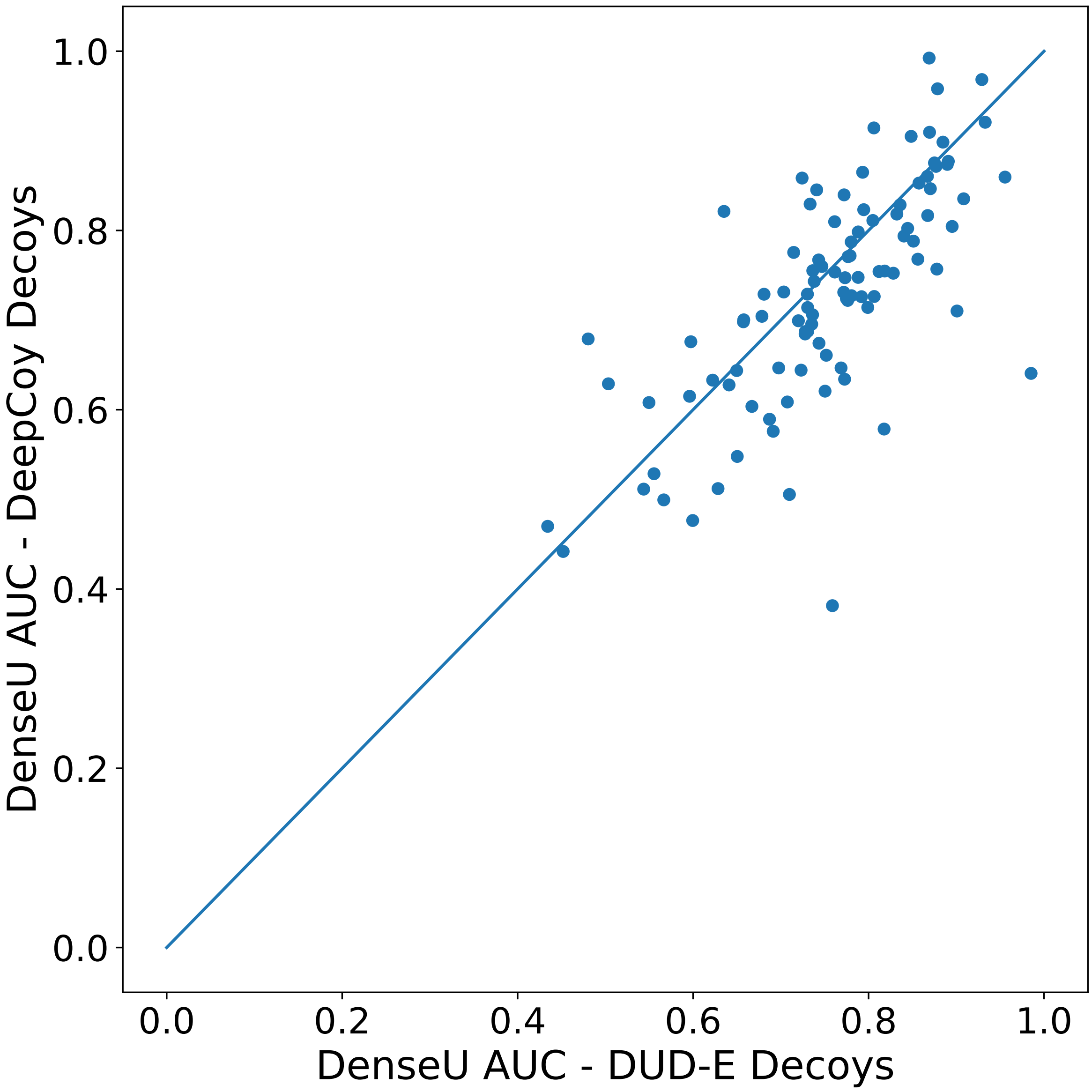

Figure S13: Virtual screening performance of DenseU[14] as measured by AUC ROC. The average AUC ROC decreased from 0.75 for the original DUD-E set to 0.73 for the DeepCoy generated decoys.

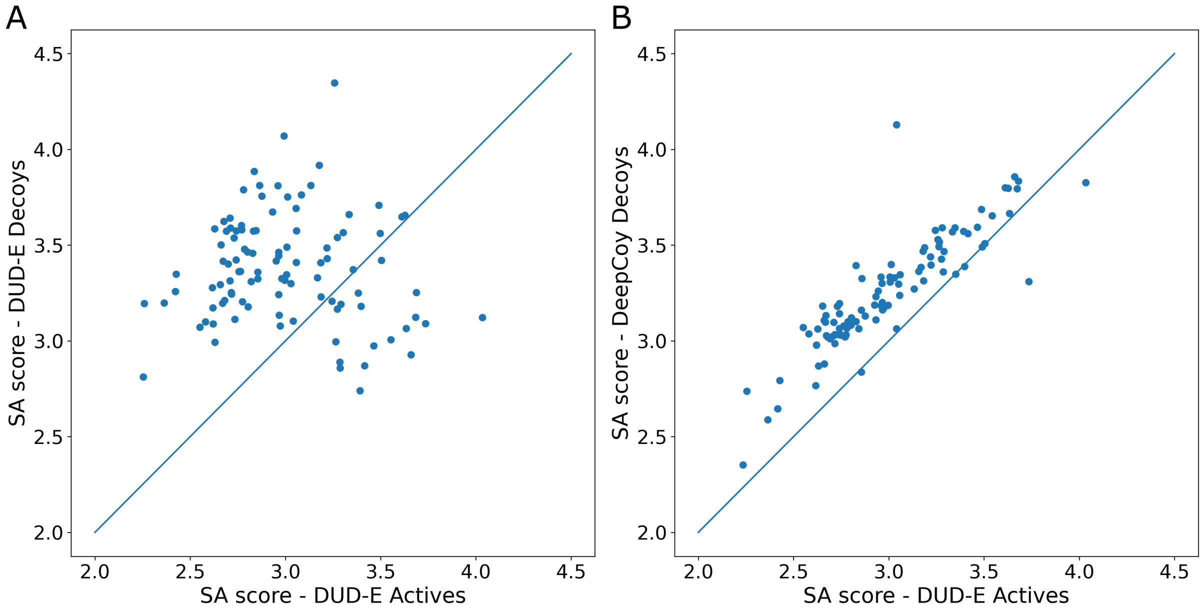

Figure S14: Average per-target Synthetic accessibility (SA) score of the original DUD-E decoys (A) and the DeepCoy decoys (B) compared to the actives. The average SA score of DeepCoy decoys is highly correlated to the actives (Pearson's R: 0.88), while there is no correlation between the original DUD-E decoys and the active molecules (Pearson's R: -0.07). In addition, the average SA score of the DeepCoy decoys (3.27) more closely matches the SA score of the active molecules (2.99) than the original decoys (3.41).

References

(1) Liu, Q.; Allamanis, M.; Brockschmidt, M.; Gaunt, A. Constrained Graph Variational Autoencoders for Molecule Design. *Advances in Neural Information Processing Systems 31 (NeurIPS)* **2018**, 7795–7804.

(2) Imrie, F.; Bradley, A. R.; van der Schaar, M.; Deane, C. M. Deep Generative Models for 3D Linker Design. *J. Chem. Inf. Model.* **2020**, *60*, 1983–1995.

(3) Sterling, T.; Irwin, J. J. ZINC 15 – Ligand Discovery for Everyone. *J. Chem. Inf. Model.* **2015**, *55*, 2324–2337.

(4) Gómez-Bombarelli, R.; Wei, J. N.; Duvenaud, D.; Hernández-Lobato, J. M.; Sánchez-Lengeling, B.; Sheberla, D.; Aguilera-Iparraguirre, J.; Hirzel, T. D.; Adams, R. P.; Aspuru-Guzik, A. Automatic Chemical Design Using a Data-Driven Continuous Representation of Molecules. *ACS Cent. Sci.* **2018**, *4*, 268–276.

(5) Li, Y.; Tarlow, D.; Brockschmidt, M.; Zemel, R. Gated Graph Sequence Neural Networks. *International Conference on Learning Representations (ICLR)* **2016**,

(6) Landrum, G. RDKit: Open-Source Cheminformatics. , [Online; accessed May 1, 2020), 2006.

(7) Mysinger, M. M.; Carchia, M.; Irwin, J. J.; Shoichet, B. K. Directory of useful decoys, enhanced (DUD-E): Better ligands and decoys for better benchmarking. *J. Med. Chem.* **2012**, *55*, 6582–6594.

(8) Bauer, M. R.; Ibrahim, T. M.; Vogel, S. M.; Boeckler, F. M. Evaluation and Optimization of Virtual Screening Workflows with DEKOIS 2.0 – A Public Library of Challenging Docking Benchmark Sets. *J. Chem. Inf. Model.* **2013**, *53*, 1447–1462.

(9) Chaput, L.; Martinez-Sanz, J.; Saettel, N.; Mouawad, L. Benchmark of four popular

virtual screening programs: construction of the active/decoy dataset remains a major determinant of measured performance. *J. Cheminf.* **2016**, *8*, 56.

(10) Rohrer, S. G.; Baumann, K. Maximum unbiased validation (MUV) data sets for virtual screening based on PubChem bioactivity data. *J. Chem. Inf. Model.* **2009**, *49*, 169–184.

(11) Ertl, P.; Schuffenhauer, A. Estimation of Synthetic Accessibility Score of Drug-Like Molecules Based on Molecular Complexity and Fragment Contributions. *J. Cheminf.* **2009**, *1*, 8.

(12) Bickerton, G. R.; Paolini, G. V.; Besnard, J.; Muresan, S.; Hopkins, A. L. Quantifying the chemical beauty of drugs. *Nat. Chem.* **2012**, *4*, 90–98.

(13) Ragoza, M.; Hochuli, J.; Idrobo, E.; Sunseri, J.; Koes, D. R. Protein-Ligand Scoring with Convolutional Neural Networks. *J. Chem. Inf. Model.* **2017**, *57*, 942–957.

(14) Imrie, F.; Bradley, A. R.; van der Schaar, M.; Deane, C. M. Protein Family-Specific Models Using Deep Neural Networks and Transfer Learning Improve Virtual Screening and Highlight the Need for More Data. *J. Chem. Inf. Model.* **2018**, *58*, 2319–2330.

(15) Bento, A. P.; Gaulton, A.; Hersey, A.; Bellis, L. J.; Chambers, J.; Davies, M.; Krüger, F. A.; Light, Y.; Mak, L.; McGlinchey, S.; Nowotka, M.; Papadatos, G.; Santos, R.; Overington, J. P. The ChEMBL bioactivity database: An update. *Nucleic Acids Res.* **2014**, *42*, 1083–1090.

(16) Riniker, S.; Landrum, G. A. Open-source platform to benchmark fingerprints for ligand-based virtual screening. *J. Cheminf.* **2013**, *5*, 26.

(17) Konc, J.; Janežič, D. ProBiS algorithm for detection of structurally similar protein binding sites by local structural alignment. *Bioinformatics* **2010**, *26*, 1160–1168.

(18) Paszke, A. et al. PyTorch: An Imperative Style, High-Performance Deep Learning Library. *Advances in Neural Information Processing Systems 32* **2019**, 8026–8037.

(19) Sunseri, J.; Koes, D. R. libmolgrid: Graphics Processing Unit Accelerated Molecular Gridding for Deep Learning Applications. *J. Chem. Inf. Model.* **2020**, *60*, 1079–1084.

(20) Sieg, J.; Flachsenberg, F.; Rarey, M. In Need of Bias Control: Evaluating Chemical Data for Machine Learning in Structure-Based Virtual Screening. *J. Chem. Inf. Model.* **2019**, *59*, 947–961.

(21) Ballester, P. J. Selecting machine-learning scoring functions for structure-based virtual screening. *Drug Discovery Today: Technologies* **2019**, *32-33*, 81 – 87.

(22) Adeshina, Y. O.; Deeds, E. J.; Karanicolas, J. Machine learning classification can reduce false positives in structure-based virtual screening. *Proc. Natl. Acad. Sci.* **2020**, *117*, 18477–18488.

(23) Koes, D. R.; Baumgartner, M. P.; Camacho, C. J. Lessons learned in empirical scoring with smina from the CSAR 2011 benchmarking exercise. *J. Chem. Inf. Model.* **2013**, *53*, 1893–1904.

(24) Trott, O.; Olson, A. AutoDock Vina: Improving the speed and accuracy of docking with a new scoring function, efficient optimization and multithreading. *J. Comput. Chem.* **2010**, *31*, 455–461.
